# A New Ship-Radiated Noise Feature Extraction Technique Based on Variational Mode Decomposition and Fluctuation-Based Dispersion Entropy

**DOI:** 10.3390/e21030235

**Published:** 2019-03-01

**Authors:** Hong Yang, Ke Zhao, Guohui Li

**Affiliations:** School of Electronic Engineering, Xi’an University of Posts and Telecommunications, Xi’an 710121, China

**Keywords:** variational mode decomposition, fluctuation-based dispersion entropy, ship-radiated noise, feature extraction

## Abstract

Sea environment complexity and underwater acoustic channels make it hard to extract features of ship-radiated noise signals. This paper presents a novel feature extraction method using the advantages of variational mode decomposition (VMD), fluctuation-based dispersion entropy (FDE) and self-organizing feature map (SOM). Firstly, VMD decomposition of the original signal is used to get a group of bandwidth-limited intrinsic mode functions (IMFs). Then, the difference between the FDE of each IMF and the original signal is calculated, respectively; the IMF with the smallest difference (SIMF) is selected to calculate the FDE as the feature vector. Finally, the characteristic vectors are sent to the SOM classifier to categorize the original signal. The proposed method is applied to feature extraction of real ship-radiated noise signals. The results show that this method is more precise for ship-radiated noise signals feature extraction.

## 1. Introduction 

Feature extraction is a key link of target classification. Sea environment complexity and underwater acoustic channels make it hard to extract features of ship-radiated noise signals that reflects the essential characteristics of the target and meet the requirements of underwater long-distance detection [1]. The generation and propagation mechanism of ship-radiated noise is complicated. Propeller cavitation is the main component of high-frequency ship-radiated noise with a high frequency continuous spectrum. At the same time, propeller beat has an obvious amplitude modulation effect on the cavitation noise of the propeller, and modulation depth is related to propeller rotation speed, number of blades, and speed. Using this feature, several inherent characteristics of the ship, such as propeller shaft frequency, blade frequency and number of blades can be effectively calculated; these radiated noise characteristics are important bases for target detection and identification [2,3]. In fact, due to the complexity and multi-path effects of underwater acoustic signals, ship-radiated noise signals tend to exhibit non-Gaussian and non-stationary properties [4]. Traditional signal processing methods assume that signals and noise are linear and stationary Gaussian random processes, such as noise envelope modulation detection (DEMOD). In the case of low signal-to-noise ratio of remote weak signals, the signal processing method based on the conventional Fourier transform finds it difficult to accurately extract the characteristics of the ship-radiated noise [5,6,7]. The Second Generation Wavelet Transform (SGWT) is a time domain transform method based on lifting strategy. This method is no longer dependent on the frequency domain and easily implements fast algorithms; at the same time, it has a good ability to suppress noise components in non-stationary signals. However, it is still affected by the wavelet basis function and decomposition level [8].

Empirical Mode Decomposition (EMD) is a new method to analyze non-stationary and non-linear signals in the signal processing field. It is a self-adaptive complete orthogonal decomposition according to the characteristics of the signal itself and can extract the eigenmode component of the dynamic signal [9,10]. Ensemble Empirical Mode Decomposition (EEMD) is a noise-assisted data analysis method based on EMD. This method improves the mode mixing of EMD, but does not have sufficient theoretical support [11,12]. In 2014, Dragomiretskiy et al. [13] proposed a new adaptive signal processing method, Variational Mode Decomposition (VMD). This method improves the disadvantages of mode mixing in the decomposition process of the EMD; compared with EEMD, VMD has enough theoretical basis. [14,15,16]. In fact, VMD is a group of multiple adaptive Wiener filters, and therefore has better robustness [17]. In recent years, with deep research on acoustic signal processing, the application of modal decomposition in underwater acoustic signals has become more mature. In [18], it is proposed to use EMD to decompose ship-radiated noise and use the characteristics of the intrinsic modal functions (IMFs) to realize feature extraction. In [19], on the basis of EMD, ship-radiated noise was classified using the center frequency of the strongest IMF of EEMD. VMD was used in [20] to diagnose wind turbine faults. Most of the above documents show that VMD is better than EMD and EEMD decomposition and can be applied to feature extraction.

Entropy is a measure of uncertainty or irregularity, first introduced by Shannon in 1948 [21]. Sample Entropy (SE) is based on Shannon entropy and is widely used in signal processing and image processing [22,23]. SE is unreliable for short time series and slow for long time sequences. Permutation Entropy (PE) is based on conditional entropy, which represents the permutation pattern or order relationship between time series amplitudes. PE is faster than SE, but considering only the order of amplitude, amplitude information may be ignored [24,25]. In order to solve the disadvantages of SE and PE, in [26], the dispersive entropy (DE) based on Shannon entropy was developed to quantify the uncertainty of time series. Since DE is a symbol-based dynamic dispersion model based on amplitude, it is also called amplitude dispersion entropy [26]. In [27], the fluctuation of the signal is used to develop Fluctuation-Based Dispersion Entropy (FDE), and to compare FDE, DE, SE, and PE, indicating that FDE and DE are superior in all aspects. In [28], PE and EMD were used to extract the characteristics of ship-radiated noise. In [29], an improved decomposition method, VMD and PE are used for fault extraction. In [30], VMD, Multi-Scale Permutation Entropy (MPE) and Support Vector Machine (SVM) were used to extract the characteristics of underwater acoustic signals. The study found that although the above method continuously improves the signal decomposition effect, it does not solve the problem that PE may lose amplitude information.

Based on the above analysis, this paper uses the advantages of VMD, FDE and SOM to propose a new method to extract ship-radiated noise characteristics. The other main contents of this paper are as follows: Section 2 describes the fundamental theories of VMD and FDE. In Section 3, the steps of the new method are briefly introduced. In Section 4, a method to analyze analog signals is used. In Section 5, the presented method is used to extract ship-radiated noise. Section 6 summarizes the full text. 

## 2. Method

### 2.1. VMD Method

VMD is a new method of modal decomposition, which introduces signal decomposition into the variational model to achieve adaptive decomposition of the signal by looking for the optimal solution of the constrained variational model, and decomposes the input signal into a series of IMF in different frequency bands [31]. VMD defined IMF as amplitude modulated frequency modulated(AM-FM) signal, such that:

(1) $$u_{k}\left(t\right)=A_{k}\left(t\right)\cos\left(\phi_{k}\left(t\right)\right)$$

where t and $A_{k}\left(t\right)$ represent time and the envelope, $\phi_{k}\left(t\right)$ and $u_{k}\left(t\right)$ denote the phase and the IMFs. 

Each IMF has a center frequency and limited bandwidth. The variational problem of VMD is to find K modal functions $u_{k}(t)$, so that the sum of all IMF estimated bandwidths is the smallest. The constraint is that the sum of the modal functions $u_{k}(t)$ is the input signal f(t). The center frequency and bandwidth of the modal function are estimated by:

(1) Performing Hilbert transform on each modal function $u_{k}(t)$ to get the analytical signal of each modal function:

(2) $$\left[\delta \left(t\right)+\frac{j}{\pi t}\right]u_{k}\left(t\right)$$

(2) Using the correction index $e^{-j\omega_{k}t}$ to modulate the spectrum of each modal function to its respective baseband:

(3) $$\left[\left(\delta \left(t\right)+\frac{j}{\pi t}\right)u_{k}\left(t\right)\right]e^{-j\omega_{k}t}$$

(3) Calculating the squared norm $L^{2}$ of the gradient of the demodulation signal in (3), and estimating the bandwidth of each modal function. The corresponding constraint variation problem is:

(4) $$\begin{array}{l} \underset{\left(u_{k}\right)\left(\omega_{k}\right)}{\min}\left\{\sum_{k=1}^{K}{\left\|\partial_{t}\left[\left(\delta \left(t\right)+\frac{j}{\pi t}\right)*u_{k}\left(t\right)\right]e^{-j\omega_{k}t}\right\|}_{2}^{2}\right\}\\ \begin{array}{cccc} & & s.t. & \sum_{k=1}^{K}u_{k}\left(t\right)=f\left(t\right) \end{array} \end{array}$$

where, s represents the processed signal, K is the number of IMFs, ∗ represent convolution. $u_{k}$ is the decomposed mono-component. $\omega_{k}$ is the center frequency for each decomposed component. $\partial_{t}$ is the inverse of the function with respect to t, δ(t) and j stand for impulse response and imaginary unit.

In order to solve the variational problem with the constraint condition in (4) into an unconstrained variational problem, the augmented Lagrangian function L is introduced as follows:

(5) $$\begin{array}{ll} L\left(\left\{u_{k}\right\},\left\{\omega_{k}\right\},\lambda \right) & =\alpha \sum_{k=1}^{K}{\left\|\partial_{t}\left[\left(\delta \left(t\right)+\frac{j}{\pi t}\right)*u_{k}\left(t\right)\right]e^{-j\omega t}\right\|}_{2}^{2}\\ & +{\left\|f\left(t\right)-\sum_{k=1}^{K}u_{k}\left(t\right)\right\|}_{2}^{2}+\langle \lambda \left(t\right),\sum_{k=1}^{K}u_{k}\left(t\right)\rangle \end{array}$$

where λ and α are the Lagrangian multiplier and balancing parameter.

Solve the extended Lagrangian function in (4) using the alternating direction multiplier algorithm. The specific steps are as follows:

(1) Initialization $\left\{{\hat{u}}_{k}^{1}\right\}$, $\left\{\omega_{k}^{1}\right\}$, ${\hat{\lambda }}^{1}$, n;

(2) Execution loop n=n+1;

(3) For ω≥0, update the fan functions ${\hat{u}}_{k}$ and $\omega_{k}$:

(6) $$\begin{array}{l} {\hat{u}}_{k}^{n+1}\leftarrow \frac{\hat{f}\left(\omega \right)-\sum_{i=1,i<k}^{K}{\hat{u}}_{i}^{n+1}\left(\omega \right)-\sum_{i=1,i>k}^{K}{\hat{u}}_{i}^{n}\left(\omega \right)+\frac{\hat{\lambda }\left(\omega \right)}{2}}{1+2\alpha {\left(\omega -\omega_{k}^{n}\right)}^{2}}\\ \begin{array}{cccc} & & & k\in \left\{1,K\right\} \end{array} \end{array}$$

(7) $$\omega_{k}^{k+1}\leftarrow \frac{\int_{0}^{\infty }\omega {\left|{\hat{u}}_{k}^{n+1}\left(w\right)\right|}^{2}d\omega }{\int_{0}^{\infty }{\left|{\hat{u}}_{k}^{n+1}\left(w\right)\right|}^{2}d\omega },k\in \left\{1,K\right\}$$

(4) Update λ:

(8) $${\hat{\lambda }}^{n+1}\left(\omega \right)\leftarrow {\hat{\lambda }}^{n}\left(\omega \right)+\tau \left[\hat{f}\left(\omega \right)-\sum_{k=1}^{K}{\hat{u}}_{k}^{n+1}\left(\omega \right)\right]$$

where ∧ and τ indicate Fourier transform and time step.

(5) Repeat steps (3)–(5) until the iteration constraint condition is satisfied:

(9) $$\sum_{k=1}^{K}\left({\left\|{\hat{u}}_{k}^{n+1}-{\hat{u}}_{k}^{n}\right\|}_{2}^{2}/{\left\|{\hat{u}}_{k}^{n}\right\|}_{2}^{2}\right)<\varepsilon$$

where ε is the accuracy for convergence. The iteration is ended, and K IMF components with the smallest sum of bandwidths are obtained.

### 2.2. FDE Method

FDE is susceptible to changes in the synchronization frequency, amplitude value, and time-series bandwidth, and does not require the ordering of the amplitude values of each embedded vector, nor the distance between adjacent embedding dimensions; thus, it is very fast [27], and also solves the problem that PE will lose amplitude information. Given a set of time series $x=x_{1},x_{2},\cdots x_{N}$ with a sequence length of N, the FDE method is introduced as follows:

(1) First, $x_{j}(j=1,2,\ldots ,N)$ are mapped to classes 2c−1 with integer indices from 1 to 2c−1 using the normal cumulative distribution function (NCDF). The classified signal is $z_{j}(j=1,2,\ldots ,N)$.

(2) Time series $z_{i}^{m,c}$ is created based on $z_{i}^{m,c}=\left\{z_{i}^{c},z_{i+d}^{c},\cdots ,z_{i+(m-1)d}^{c}\right\}$, i=1,2,⋯N−(m−1)d, where m is the embedded dimension, d is the time delay. Each time series $z_{i}^{m,c}$ is mapped to a dispersion patter $\pi_{v_{0}v_{1}\cdots v_{m-1}}$, where $z_{i}^{c}=v_{0},z_{i+d}^{c}=v_{1},\cdots ,z_{i+(m-1)d}^{c}=v_{m-1}$. The number of possible dispersion patterns assigned to each vector $z_{i}^{m,c}$ is equal to ${\left(2c-1\right)}^{m-1}$. since the signal $z_{i}^{m,c}$ has m elements and each can be one of the integers from −c+1 to c−1.

(3) For each potential dispersion mode ${\left(2c-1\right)}^{m-1}$, the relative frequency is:

(10) $$p(\pi_{v_{0}v_{1}\cdots v_{m-1}})=\frac{\#\left\{i|i\leq N-(m-1)d,z_{i}^{m,c}\;has\;type\;\pi_{v_{0}v_{1}\cdots v_{m-1}}\right\}}{N-(m-1)d}$$

where, # means cardinality, in fact, $p(\pi_{v_{0}v_{1}\cdots v_{m-1}})$ reveals that the number of distribution models $\pi_{v_{0}v_{1}\cdots v_{m-1}}$ allocated to $z_{i}^{m,c}$, divided by the gross number of embedded signals, embedded dimension of which is m.

(4) Lastly, according to the calculation methods of entropy, the FDE is calculated as follows:

(11) $$FDE(x,m,c,d)=-\sum_{\pi =1}^{{\left(2c-1\right)}^{m-1}}p(\pi_{v_{0}v_{1}\cdots }{}_{v_{m-1}})\cdot \ln(p(\pi_{v_{0}v_{1}\cdots }{}_{v_{m-1}}))$$

where m is the embedded dimension, d is the time delay, and c is the class number.

As an example, let us have a signal x={1.2,5.5,3.9,5.8,4.1,6.5,2.2,4.9,5.6,7.8}. We set d=1, m=3, c=2, leading to $3^{2}=9$ potential fluctuation-based dispersion patterns, ({(−1,−1),(−1,0),(−1,1),(0,−1),(0,0),(0,1),(1,−1),(1,0),(1,1)}). Then, $x_{j}\left(j=1,2,\ldots ,10\right)$ are linearly mapped into two classes with integer indices from 1 to 2 ({1,1,2,2,1,1,1,2,2,2}). Afterwards, a window with length 3 moves along the time series and the differences between adjacent elements are calculated x={(0,1),(1,0),(0,−1),(−1,0),(0,0),(0,1),(1,0),(0,0)}. Afterwards, the number of each fluctuation-based dispersion pattern is counted. Finally, using Equation (11), the DispEn value of x is $-\left(\frac{3}{8}\ln\left(\frac{3}{8}\right)+\frac{3}{8}\ln\left(\frac{3}{8}\right)+\frac{1}{8}\ln\left(\frac{1}{8}\right)+\frac{1}{8}\ln\left(\frac{1}{8}\right)\right)=1.2555$.

In order to test the advantages of FDE, the PE, MPE, and FDE of simulation signal cos(10πt) were calculated. The data lengths are 1000/2000/3000, respectively; the results are shown in Table 1. The PE, MPE, and FDE of simulation signal cos(2πft) were calculated—the data length is 1000, the frequency f is 10/50/80, respectively; the results are shown in Table 2. The sampling frequency is set as 1000 Hz. In Table 1, the signal frequency is unchanged, and the corresponding entropy changes with the change of the signal length; the change of FDE value is the smallest. In Table 1, the signal length remains unchanged, and the PE MPE and FDE values change with the change of signal frequency. In Table 2, the signal length remains unchanged, and the PE MPE and FDE values change with the change of signal frequency. As seen in Table 1 and Table 2, the change of FDE value is the smallest and stability of FDE is the best. In summary, FDE is more stable and more suitable for feature extraction.

### 2.3. Test with the Analog Signals Using VMD and FDE

**Figure 1:**
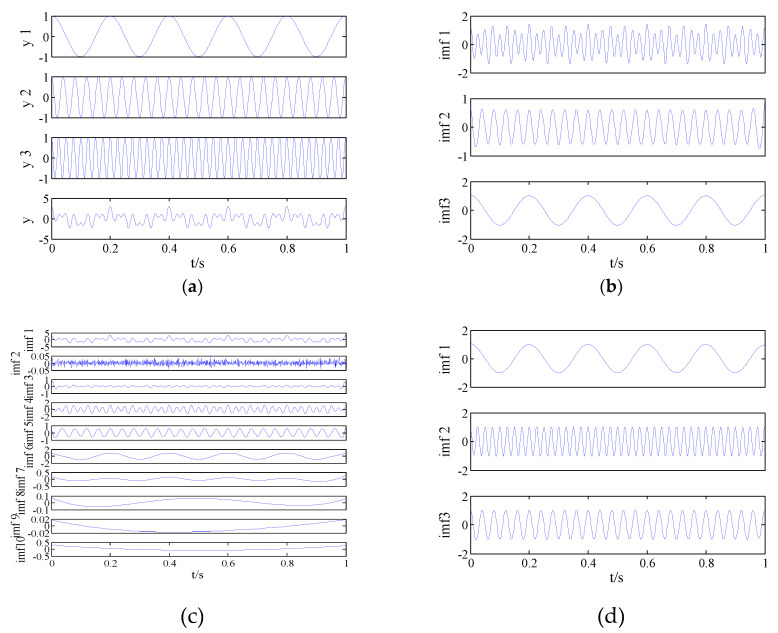
Original signal and decomposition results of EMD, EEMD, and VMD. (**a**) Original signal; (**b**) EMD result; (**c**) EEMD result; and (**d**) VMD result.

To further illustrate the advantages of VMD algorithm, the analog signal is decomposed by EMD, EEMD, and VMD in this section. The analog signal is as follows:

(12) $$\left\{ \begin{array}{l} y1=cos\left(10\pi t\right)\\ y2=cos\left(50\pi t\right)\\ y3=cos\left(80\pi t\right)\\ y=y1+y2+y3 \end{array}\right.$$

where y1, y2, y3 are the three components of y, and the sampling frequency of the analog signal is 1 kHz. The original signal is shown in Figure 1a. The EMD decomposition result is shown in Figure 1b. The white noise added during EEMD decomposition is 0.2, and the number of iterations is 200; the decomposition result is shown in Figure 1c. The optimal number of VMD decompositions is 3, the step size is 0.03, the tolerance is $1e^{-7}$, the penalty parameter is 2000, and the decomposition result is shown in Figure 1d. As seen in Figure 1b, there is a serious mode mixing phenomenon in the IMF1. In Figure 1c, the mode mixing is improved, but the added white noise has a great influence on the original signal. In Figure 1d, there is no mode mixing and there is no noise effect. In order to further compare the three modal decompositions, the error between the components of the analog signal and the corresponding IMF is calculated. Table 3 shows that y1, y2, and y3 correspond to IMF1, IMF2, and IMF3 after the VMD decomposition. At this time, error is the smallest and decomposition effect is the best.

In order to highlight the differences between PE, MPE, and FDE, the analog signal formula (12) is analyzed to calculate the components of the analog signal and the corresponding IMF’s PE, MPE, and FDE; the results are show in Table 4. As observed, the difference between the FDE and analog signal components under VMD decomposition is the smallest, which can better reflect the characteristics of the original signal.

## 3. Feature Extraction Technique Using VMD and FDE

The basic theory of VMD and FDE and their respective advantages were introduced in Section 2. In recent years, VMD has been more widely used in feature extraction. However, as a newly proposed algorithm, FDE has few practical applications and is not used in feature extraction. Therefore, a new feature extraction technique using VMD and FDE is proposed. Specific steps are as follows:

(1) Determine the optimal decomposition number of VMD
(2) The signal is decomposed by VMD, and the intrinsic mode functions are obtained
(3) Calculate the FDE of the original signal and IMF, respectively
(4) Calculate the FDE difference between the IMF and the original signal
(5) The IMF with the smallest FDE difference is selected as the optimal IMF, denoted as SIMF
(6) The FDE of the SIMF is entered into the SOM to observe the classification results

## 4. Simulation

In order to verify the effectiveness of the proposed method in Section 3, feature extraction is performed on three typical chaotic signals—Lorenz chaotic system, Rossler chaotic system and Duffing chaotic system. When given appropriate parameters, three systems have chaotic characteristics.

The Lorenz system can be expressed as:

(13) $$\begin{array}{l} \dot{x}=-\sigma (x-y)\\ \dot{y}=-xz+rx-y\\ \dot{z}=xy-bz \end{array}$$

where σ=10, b=8/3, r=28,$\left[\begin{array}{ccc} x\left(0\right) & y\left(0\right) & z\left(0\right) \end{array}\right]=\left[\begin{array}{ccc} 0 & 0 & 0 \end{array}\right]$.

The Rossler system can be expressed as:

(14) $$\begin{array}{l} \dot{x}=-y-z\\ \dot{y}=x+ay\\ \dot{z}=xz-cz+b \end{array}$$

where a=0.2, b=0.2, c=5.7,$\left[\begin{array}{ccc} x\left(0\right) & y\left(0\right) & z\left(0\right) \end{array}\right]=\left[\begin{array}{ccc} 0 & 0 & 0 \end{array}\right]$.

The Duffing system can be expressed as:

(15) $$\begin{array}{l} \dot{x}=y\\ \dot{y}=-bx+ay-y^{3} \end{array}$$

where a=0.82, b=−0.5,$\left[\begin{array}{ccc} x\left(0\right) & y\left(0\right) & z\left(0\right) \end{array}\right]=\left[\begin{array}{ccc} 0 & 0 & 0 \end{array}\right]$.

**Figure 2:**
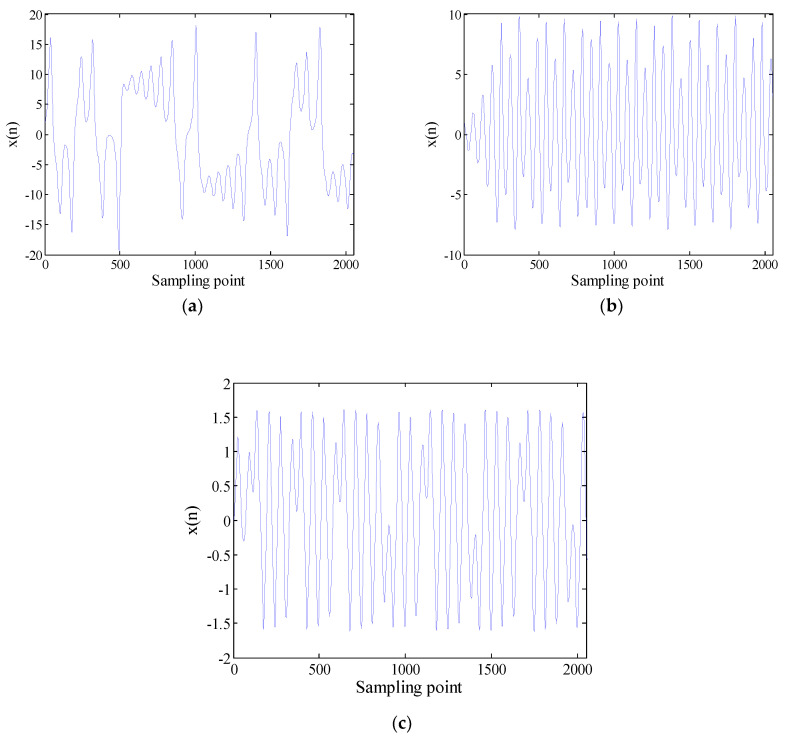
Timing diagrams of (**a**) Lorenz, (**b**) Logistic, and (**c**) Duffing.

**Figure 3:**
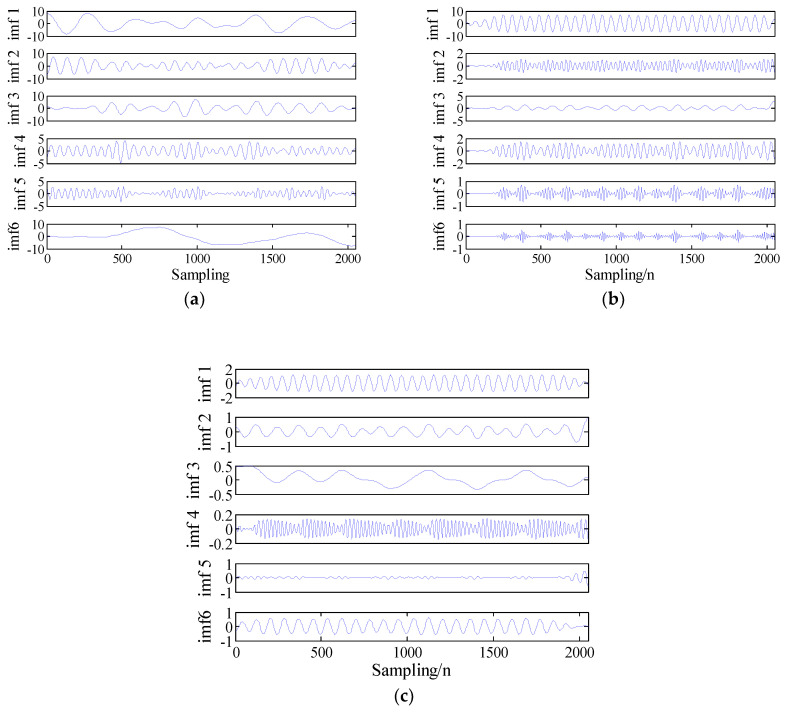
Results of VMD for (**a**) Lorenz, (**b**) Logistic, and (**c**) Duffing.

**Figure 4:**
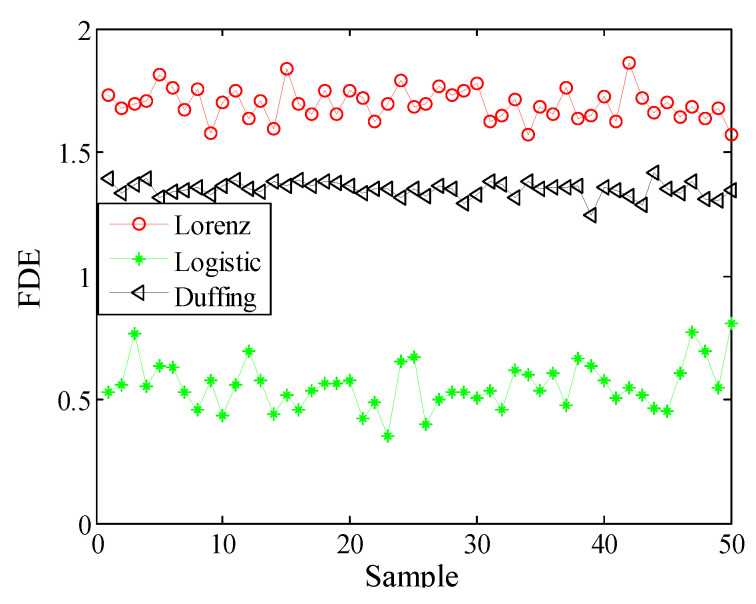
FDEs of Lorenz, Logistic and Duffing.

These equations are integrated by using a fourth-order Runge-Kutta method with a fixed step size of 0.01. The x component signal with a length of 2048 points is selected as a chaotic signal. These time-domain waveforms for Lorenz, Logistic and Duffing are shown in Figure 2. Analyze chaotic signals according to the steps described in Section 3. The decomposition results of the VMD for chaotic signals are presented in Figure 3. The difference between the chaotic signal and their IMF’s FDE is as shown in Table 5. From Table 5, we can see that the SIMF of Lorenz, Logistic and Duffing are IMF5, IMF1 and IMF5, respectively. Select 50 sampling points randomly in the SIMF and calculate FDE as the feature vector. The FDE values of the three chaotic signals are shown in Figure 4. It can be clearly seen that the three kinds of signals are clearly distinguished, which verifies the reliability of the proposed method. The ship-radiated noise signal is a chaotic signal; this method can be used to extract its features. 

## 5. Feature Extraction of Ship-Radiated Noise Using VMD and FDE

### 5.1. Analysis of Ship-Radiated Noise Using VMD

**Figure 5:**
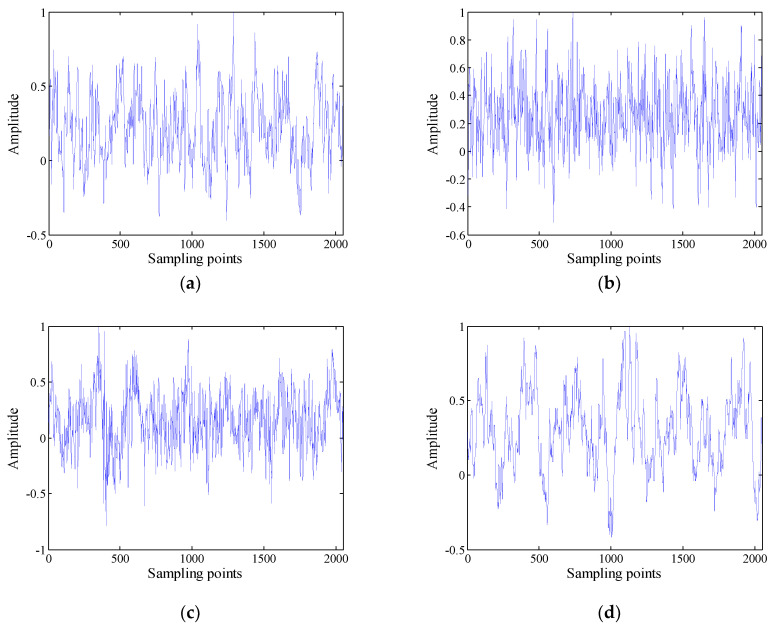
Radiated noise of four types of ships: (**a**) First, (**b**) second, (**c**) third, (**d**) fourth.

**Figure 6:**
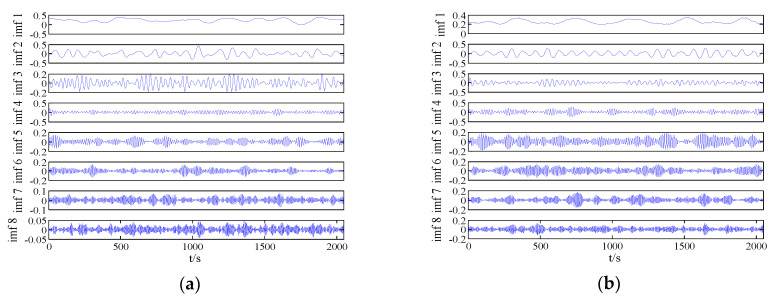

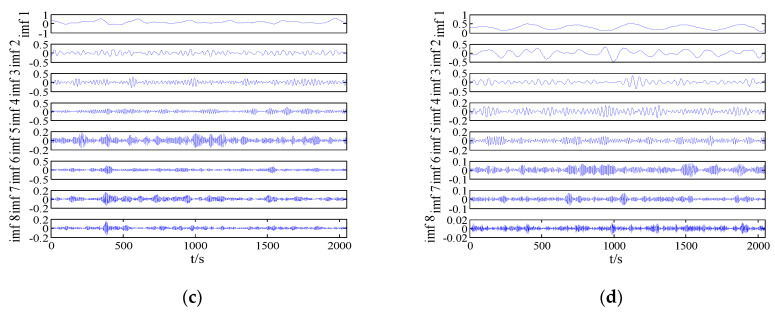
Results of VMD for radiated noise of four types of ships: (**a**) First, (**b**) second, (**c**) third, (**d**) fourth.

The data used in this paper are four different kinds of actual ship-radiated noise measured in a sea area of South China Sea and under the same conditions. Each type of ship-radiated noise has a certain amount of sample data, with a sample length of 2048 points and a sampling frequency of 20 kHz. The ship-radiated noise is normalized, as shown in Figure 5. The normalized ship-radiated noise is decomposed into IMF using VMD. After continuous testing, it can be seen that when the optimal decomposition number K>8, the subsequent IMF tends to be similar, so the optimal decomposition number K is 8, the step length is 0.03, the tolerance is $1e^{-7}$, and the penalty parameter is 2000, as shown in Figure 6.

### 5.2. Feature Extraction of Ship-Radiated Noise

According to the VMD decomposition results of the ship-radiated noises in Section 5.1, the FDE difference of the original ship-radiated noises and each IMF was calculated to obtain SIMF, as shown in Table 6. It can be seen from Table 6 that the SIMFs corresponding to ship-radiated noises are respectively IMF4, IMF5, IMF4 and IMF3.

**Figure 7:**
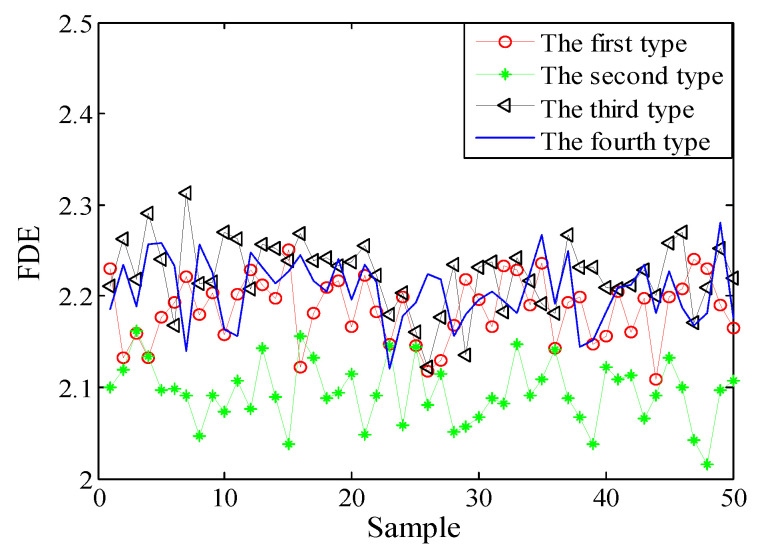
FDEs of four types of ship-radiated noises.

**Figure 8:**
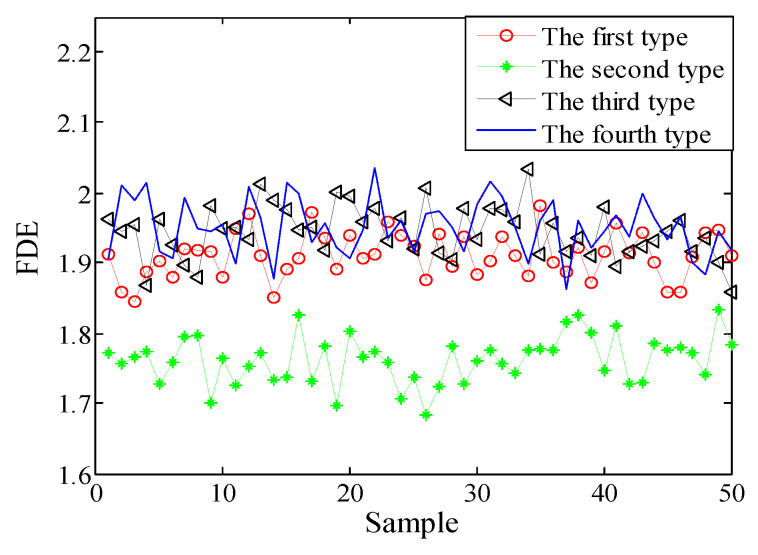
FDEs of IMF with the highest CC by VMD.

**Figure 9:**
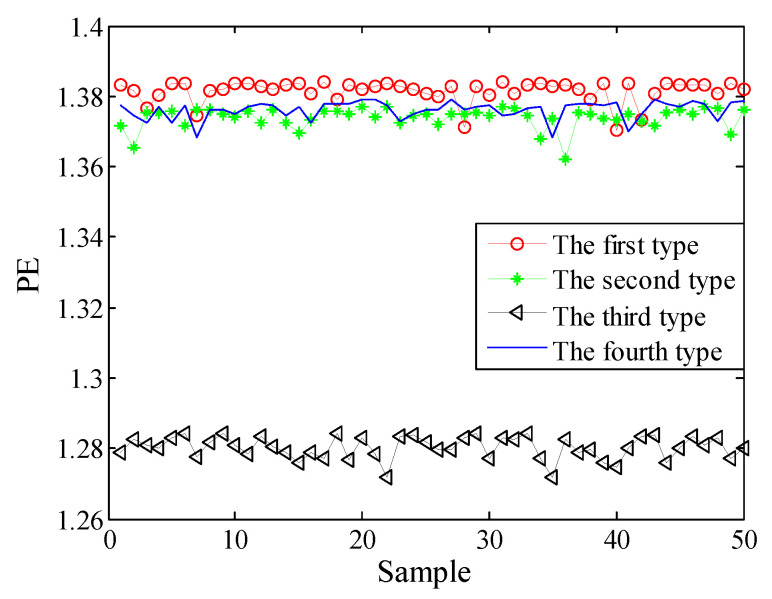
PEs of the SIMF by VMD.

**Figure 10:**
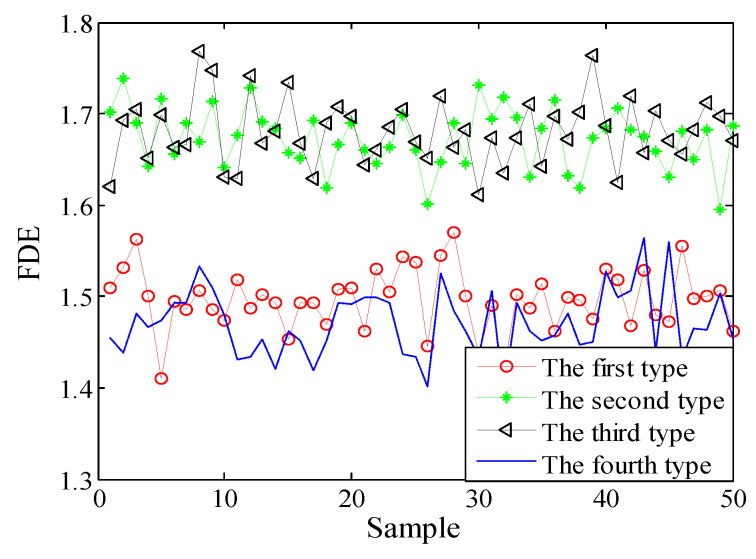
FDEs of the SIMF by EMD.

**Figure 11:**
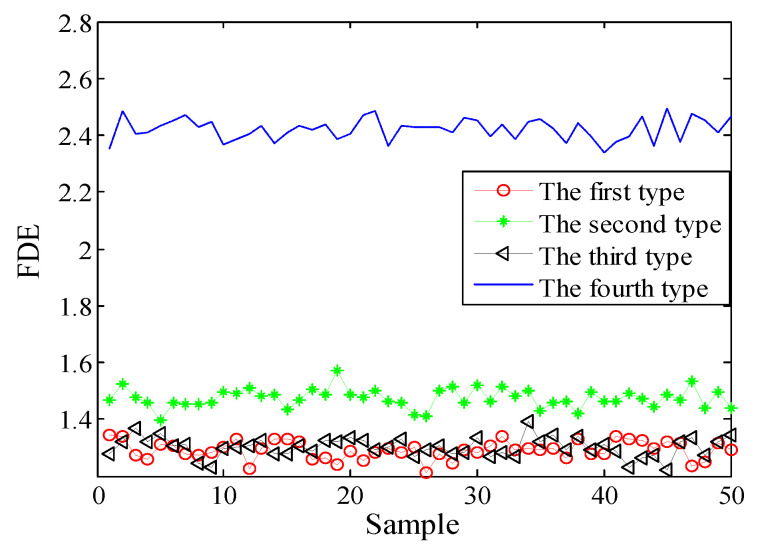
FDEs of the SIMF by EEMD.

**Figure 12:**
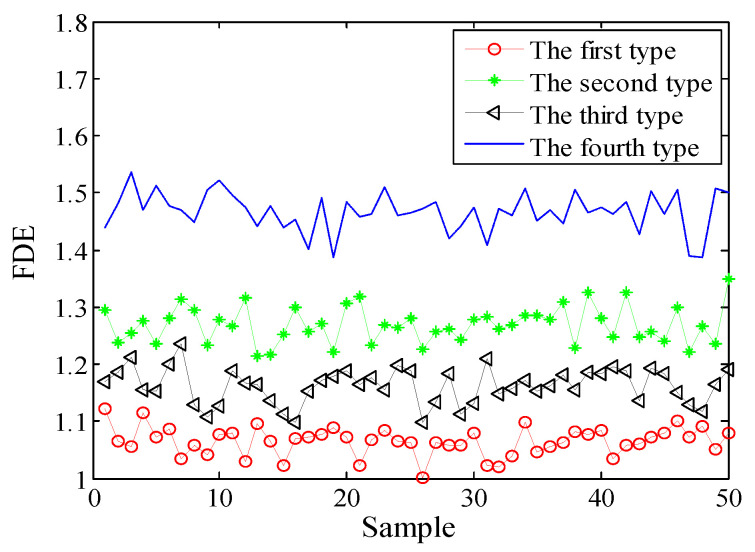
FDEs of the SIMF by VMD.

Selecting 50 sampling points randomly in the ship-radiated noises, calculate FDE as the feature vector. The FDE values of the four kinds of ship-radiated noises are shown in Figure 7. Because of the influence of ocean background noise, it is impossible to effectively distinguish the four kinds of ship-radiated noises. In order to compare with the method proposed in this paper, the FDEs with the highest correlation coefficient (CC) and the PEs of the SIMF after VMD decomposition of the four types of ship-radiated noises are calculated, as shown in Figure 8 and Figure 9. In Figure 8, although the second type of ship can be distinguished, the other three are indistinguishable. In Figure 9, four kinds of ship-radiated noises are inseparable. To obtain SIMFs, the four types of ship-radiated noises are decomposed by EMD, EEMD, and VMD and respectively calculate FDE. Each SIMF selects 50 sampling points randomly to calculate the FDE. The results are shown in Figure 10, Figure 11 and Figure 12. As seen in Figure 10, the first type and the fourth type are at the same level and cannot be distinguished. The second type and the third type are at the same level and cannot be distinguished. In Figure 11, the first and third type cannot be distinguished. In Figure 12, it can be clearly seen that the four types of ships are clearly distinguished. Therefore, the method proposed in this article is the best.

### 5.3. Classification of Ship-radiated Noise

Section 5.2 proves the effectiveness of the proposed technique by observing the distribution of FDE. The results of Section 5.2 are sent into the SOM classifier for classification and identification. The first 25 data are selected as training samples and the last 25 as test samples. The classification results are shown in Table 7. As seen in Table 7, the error rate of the EMD-SIMF-FDE method is the highest; it is more than 44%. The proposed method in this paper is only 2.5%; the error rate of the fourth type of ship-radiated noises is zero. This results show that the method proposed in this paper is the best.

## 6. Conclusions

In order to achieve feature extraction of ship-radiated noises, a new method combining VMD and FDE is proposed to distinguish different types of ship-radiated noises. The four types of ship-radiated noises are decomposed by using VMD, and the IMF with the smallest FDE difference is obtained. Then, the FDE of the IMF with the smallest FDE difference is calculated, and the FDE sent into a SOM classifier to classify the four types of ships.

(1) The VMD algorithm is a new adaptive signal processing method, which solves the mode mixing and end effects of EMD and EEMD. The simulation results show that VMD decomposition is better and more conducive to feature extraction of signals.
(2) FDE is a new type of entropy, which solves the disadvantage that PE only considers the sequence of amplitude and may lose the message of amplitude. The simulation results in Section 2.3 show that FDE is more stable than PE and MPE and is more suitable for feature extraction.
(3) In this paper, VMD, FDE and SOM are combined for feature extraction at the first time. The method proposed in this paper is compared with the IMF with the highest correlation coefficient after VMD decomposition and SIMF after EMD and EEMD decomposition. The results show that the presented technique has better separation effect and higher discrimination.

## Figures and Tables

**Table 1:** PE, MPE and FDE with different lengths and same frequency.

Data Length	1000	2000	5000
PE	0.8065	0.8114	0.8135
MPE	0.8738	0.8814	0.8859
FDE	0.1958	0.1957	0.1957

**Table 2:** PE, MPE and FDE with same length and different frequencies.

Frequency	10	50	80
PE	0.8065	1.1692	1.5538
MPE	0.8738	1.3263	1.7834
FDE	0.1958	0.5394	0.8491

**Table 3:** Errors between the components of the analog signal and the corresponding IMF.

	EMD	EEMD	VMD
*y*1	IMF3 0.0219	IMF6 0.2272	IMF1 0.0027
*y*2	IMF2 0.2527	IMF5 0.2939	IMF2 0.0031
*y*3	IMF1 0.2512	IMF4 0.0819	IMF3 0.0033

**Table 4:** PE, MPE, FDE of each component of the analog signal and the corresponding IMF.

		Analog Signal	EMD	EEMD	VMD
	PE	0.8065	0.8082	0.8072	0.8027
*y*1	MPE	0.8738	0.8838	0.9012	0.8738
	FDE	0.1958	0.1958	0.1958	0.1958
	PE	1.1492	1.1662	1.1631	1.1476
*y*2	MPE	1.3263	1.4126	1.4569	1.3263
	FDE	0.6394	0.6394	0.6394	0.6394
	PE	1.3538	1.3560	1.3556	1.3492
*y*3	MPE	1.7834	1.7573	1.7511	1.7591
	FDE	0.8491	0.8231	0.8099	0.8491

**Table 5:** Difference between the chaotic signals and their IMF’s FDE.

	IMF1	IMF2	IMF3	IMF4	IMF5	IMF6
Lorenz	0.4338	0.2261	0.3391	0.0764	0	0.5135
Logistic	0.0026	0.2383	0.1780	0.1035	0.3320	0.4517
Duffing	0.0440	0.1519	0.3197	0.4372	0.0246	0.0424

**Table 6:** IMFs with the smallest FDE difference between the four types of ship-radiated noises.

First Type		Second Type		Third Type		Fourth Type	
IMF4	0.1085	IMF5	0.0542	IMF4	0.075	IMF3	0.1111

**Table 7:** The classification results of different methods.

Method	Numbers of Errors				Error
	First Type	Second Type	Third Type	Fourth Type	Ratio (%)
EMD-SIMF-FDE	18	24	28	18	44
EEMD-SIMF-FDE	24	1	22	0	23.5
VMD-CC-FDE	16	1	30	16	31.5
VMD-SIMF-PE	5	4	0	50	29.5
VMD-SIMF-FDE	1	1	3	0	2.5
